# The risk of suicide in a group of men hospitalized in forensic psychiatry departments

**DOI:** 10.3389/fpsyt.2025.1752028

**Published:** 2026-01-16

**Authors:** Joanna Fojcik, Michał Górski, Justyna Szulik, Edyta Bogunia, Rafał Skowronek, Marek Krzystanek

**Affiliations:** 1 Department of Psychiatry, Department of Neurology, Faculty of Health Sciences in Katowice, Medical University of Silesia in Katowice, Katowice, Poland; 2 Department of Chronic Diseases and Civilization Threats, Department of Occupational Medicine and Hygiene, Faculty of Public Health in Bytom, Medical University of Silesia in Katowice, Bytom, Poland; 3 Department of Histology and Embryology, Department of Cytophysiology, Faculty of Medical Sciences, Medical University of Silesia in Katowice, Katowice, Poland; 4 Department of Forensic Medicine and Forensic Toxicology, Faculty of Medical Sciences in Katowice, Medical University of Silesia in Katowice, Katowice, Poland; 5 Department and Clinic of Psychiatric Rehabilitation in Katowice, Faculty of Medical Sciences in Katowice, Medical University of Silesia in Katowice, Katowice, Poland; 6 Department of Clinical and Community Psychiatry and Psychology Collegium Medicum, WSB University, Dąbrowa Górnicza, Poland

**Keywords:** forensic psychiatry, isolation, risk of suicide, suicidal ideation, suicidal tendencies

## Abstract

**Purpose:**

The aim of this study was to determine the risk of suicidal behavior in patients who were involuntarily hospitalized in forensic psychiatry departments, taking into account demographic variables such as age, level of education and length of stay of patients in the department.

**Material and methods:**

The study involved 112 patients of forensic psychiatry wards with a basic level of security, who completed an original questionnaire covering demographic data and the SBQ-R questionnaire on suicidal behavior.

**Results:**

The vast majority of patients studied were assigned to the low-risk category (96%), meaning they did not exhibit significant symptoms suggesting suicidal risk. Statistical analysis revealed no significant relationship between patient age and suicidal risk. Similarly, length of hospitalization had no significant effect on SBQ-R scores.

**Conclusions:**

Most patients scored low on the Suicide Risk Assessment Scale (SBQ-R), indicating a low level of suicidal ideation and tendencies.

## Introduction

It is estimated that as many as 90% of people who decide to commit suicide had at least one psychiatric diagnosis. Individuals diagnosed with schizophrenia, depression, and addiction are particularly at risk of suicide (1, 2).

Suicide is a multidimensional, complex phenomenon, with varying definitions. One definition, adopted by the WHO, defines suicide as the act of intentionally taking one’s own life. Similarly, suicidal behavior is most often defined as potentially fatal actions undertaken with the intention of taking one’s own life. Suicide attempts are usually considered to be those accompanied by a clearly expressed suicidal intent or those that involve a significant threat to life. A key element of these definitions is their premeditation and intentionality. Suicidal ideation, understood as the desire to take one’s own life, usually precedes suicide attempts, including suicides (3).

In everyday clinical practice in psychiatry, many people suffering from mental disorders attempt suicide. Due to the specific nature of suicide, estimation of risk is based on indirect risk factors, such as suicidal thoughts and tendencies. It is often noted that patients with a current or past history of suicidal behavior are at particularly high risk (2).

A closed psychiatric facility implementing security measures focuses primarily on ensuring social safety, followed by therapy and rehabilitation. Individuals ordered by the court to so-called judicial detention remain there as long as their mental health and behavior pose a risk of reoffending in a particularly harmful crime. Release from detention, which may occur when the risk of recurrence of threatening behavior ceases, results primarily from treatment, not isolation itself, and is often only the beginning of a long-term therapeutic process outside the psychiatric hospital (4).

Due to the lack of Polish research on suicide risk assessment in patients admitted to forensic psychiatry wards, we decided to conduct such a study. The conclusions drawn from this study may influence daily clinical practice and determine the need to implement special procedures to ensure the safety of these patients.

## Materials and methods

The study was conducted among 112 patients of forensic psychiatry wards. The main aim of the study was to assess the risk of suicide among patients involuntarily hospitalized in forensic psychiatry wards with a basic level of security. The study used a demographic and clinical survey consisting of 8 questions concerning the patient’s personal details, diagnosis, and length of stay in the ward. The Suicide Behavior Questionnaire (Suicide Behaviors Questionnaire-Revised (SBQ-R), which in its 4 questions allows to shed light on the patient’s past towards suicidal tendencies, and also paints a picture of whether a given person may have suicidal thoughts in the future.

### Data analysis methodology

Statistical analysis of the obtained results was performed using MS Excel spreadsheet and specialized Statistica software, version 13 (TIBCO Software Inc., 2017).

Data for analysis were collected as part of a diagnostic survey study involving 112 patients (N = 112) involuntarily hospitalized in basic- security forensic psychiatric wards. This sample allows for the assessment of the variables studied in the context of this patient group. The study utilized two key diagnostic tools:

Sociodemographic questionnaire – contained questions about basic patient data, such as age, gender, marital status, place of residence, level of education, professional activity or source of income, length of stay in the ward, psychiatric diagnosis and previous suicide attempts.

Behavior Questionnaire (SBQ-R) – a tool for assessing the risk of suicidal behavior, taking into account both patients’ previous experiences and current suicidal tendencies.

Suicide Behavior Questionnaire​ Behaviors Questionnaire-Revised (SBQ-R) consists of four questions that concern:

− History of suicidal thoughts and attempts (whether and how often they occurred in the past).
− Frequency of suicidal thoughts in the last year.
− The predicted likelihood of committing suicide in the future.

The SBQ-R results are interpreted according to the total score as follows:

− Low risk level: 3–7 points (monitoring and psychoeducation recommended).
− Medium risk level: 8–11 points (psychological consultation and therapeutic support recommended).
− High risk level: 12–18 points (urgent psychiatric intervention and specialist evaluation recommended).

Clinical suicide risk is assumed for the following thresholds:

≥ 7 points in the general population suggests an increased risk.
≥ 8 points in the clinical population indicates a significant risk of suicide.

Due to the fact that the initial analysis indicated a non-normal distribution, a nonparametric statistical method was used to verify the significance of differences between selected variables: the Kruskal- Wallis test. The adopted level of statistical significance was α = 0.05, meaning that differences were considered statistically significant if the p value was lower than 0.05.

### Characteristics of the study group

The study involved 112 patients in basic-security forensic psychiatry wards. The study group consisted exclusively of men, with an average age of 47, with the youngest patient being 24 and the oldest 85. Regarding psychiatric diagnoses, schizophrenia was by far the most common (73% - 82). Depressive disorders (5% - 6) and anxiety disorders (4% - 5) were significantly less common. A diagnosis of bipolar disorder (BD) was made in 4% (4) of the study participants, and 13% (15) of the patients had other psychiatric diagnoses. Single men predominated among the study participants, with married individuals constituting 10%. Before hospitalization, 52% of respondents lived with their family, 39% of respondents lived alone, 9% of respondents stayed in care facilities, such as Nursing and Treatment Centers or Social Welfare Homes, and their main source of income was a disability pension (49% of respondents).

Demographic analysis showed that:

− 37% of the respondents stayed in the ward for 1 to 3 years.
− 29% of patients stayed in detention for less than 12 months.
− stay from 3 to 5 years, concerned 15% of people.
− stay from 5 to 9 years – 15%.
− over 9 years - 10%.

Data obtained during the study indicate that 18% of hospitalized patients had previously attempted suicide. 82% of patients had no history of such behavior.

## Results

On the SBQ-R question regarding history of suicidal thoughts and attempts, the mean score was 1.14 with a median of 1, indicating that most patients had never had suicidal thoughts or attempted suicide. Scores ranged from 1 to 3, indicating that some patients had experienced suicidal thoughts several times, but none reported attempting suicide (none scored 4). The relatively low standard deviation (0.44) indicates that the results were quite uniform, with most patients responding similarly.

For the SBQ-R question regarding frequency of suicidal thoughts in the past year, the mean was 1.09 and the median was 1, indicating that most participants had not had suicidal thoughts in the past year. Scores ranged from 1 to 3, suggesting that there were individuals in the group who occasionally (1–2 times per year) experienced such thoughts, but no one indicated more frequent suicidal thoughts. The scale for this question ranged from 1 (no suicidal thoughts) to 5 (almost daily suicidal thoughts), and no scores above 3 meant that no one in the study group reported intense suicidal thoughts. The low standard deviation (0.37) indicates that most patients responded similarly.

For the question regarding the predicted likelihood of committing suicide in the future, the mean score was 1.04, with a median of 1, indicating that most patients did not plan to attempt suicide in the future. Scores in this category ranged from 1 to 3, but the lowest values predominated. The lowest standard deviation of all categories (0.28) suggests that patients were consistent in their responses. No scores above 3 indicate that no patients assessed their risk as high.

In the final SBQ-R question, regarding the degree of communicating suicidal thoughts to others, the greatest differences between patients were observed in the extent of communicating suicidal thoughts to others. The mean was 0.33, but the range of scores was much wider, from 0 to 6. A median of 0 indicates that most patients had never disclosed their suicidal thoughts. The highest standard deviation (0.88) among all categories indicated that patients varied greatly in their responses.

The mean SBQ-R total score in the study population was 3.61, indicating an overall low level of suicidal risk in this group. A median of 3 indicates that at least half of the patients achieved the minimum score, suggesting that a significant portion of the population does not exhibit significant symptoms of suicidal ideation or tendencies. The range of scores was 3 to 12, suggesting the presence of a small group of patients with elevated scores requiring more detailed clinical evaluation. The standard deviation (1.42) indicates relatively little variation in scores, confirming the predominance of low scores, with individual cases with significantly higher scores. The SBQ-R results are presented in **Table 1**.

**Table 1:** Suicidal Behavior Questionnaire (SBQ-R).

Variable	Descriptive statistics					
	N	Mean	Median	Min.	Max	Std Dev​
SBQ-R - 1 - History of Suicidal Thoughts and Attempts	112	1.14	1	1	3	0.44
SBQ-R - 2 - Frequency of suicidal thoughts in the last year	112	1.09	1	1	3	0.37
SBQ-R - 3 - Predicted probability of committing suicide in the future	112	1.04	1	1	3	0.28
SBQ-R - 4 - Degree of communicating suicidal thoughts to others	112	0.33	0	0	6	0.88
SBQ-R - total score	112	3.61	3	3	12	1.42

**Figure 1:**
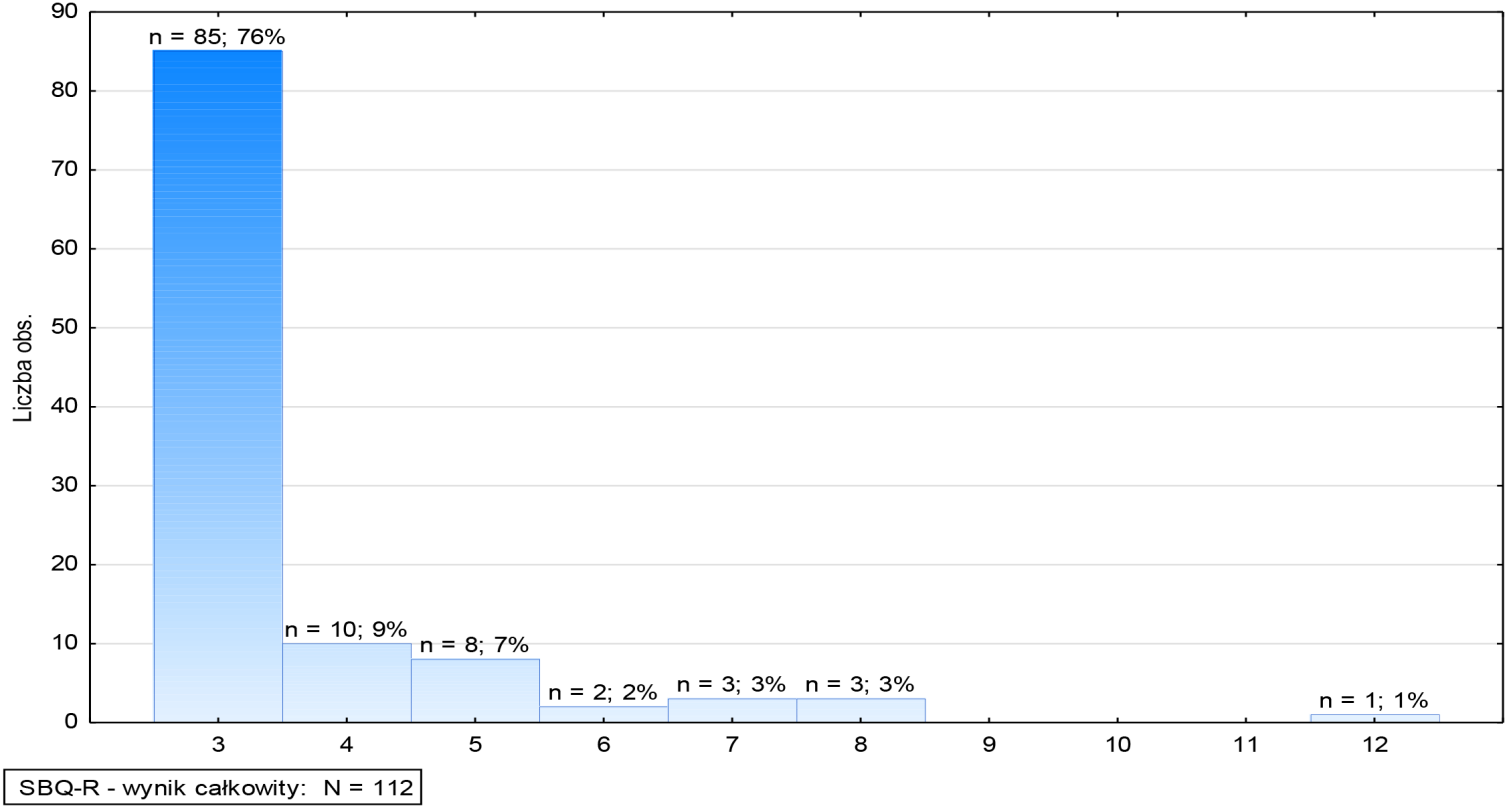
Suicidal Behavior Questionnaire (SBQ-R) – total score.

Analysis of the distribution of the SBQ-R total score (**Figure 1**) reveals a predominance of minimal values, indicating a low level of declared suicidal risk among patients involuntarily hospitalized in forensic psychiatry wards. The most common score (76%–85) was 3, indicating that the vast majority of patients did not exhibit significant symptoms of suicidal ideation or behavior. A score of 4 was achieved by 9% (10 subjects), while a score of 5 was achieved by 7% (8 subjects). Higher scores were rare – 2% (2 subjects) achieved a score of 6, and 3% (6 subjects in total) achieved scores of 7 and 8. The maximum score of 12 was achieved by only one respondent (1%), representing the highest risk level in the analyzed group.

Analysis of the clinical risk threshold revealed the presence of individual patients with scores above 8 points (4%–4), indicating the existence of a group at potentially increased risk, requiring further diagnostics and individual clinical assessment (**Figure 1**). However, 96% (108) of patients did not meet the clinical risk criteria, suggesting that the risk of suicide in this population is limited.

**Figure 2:**
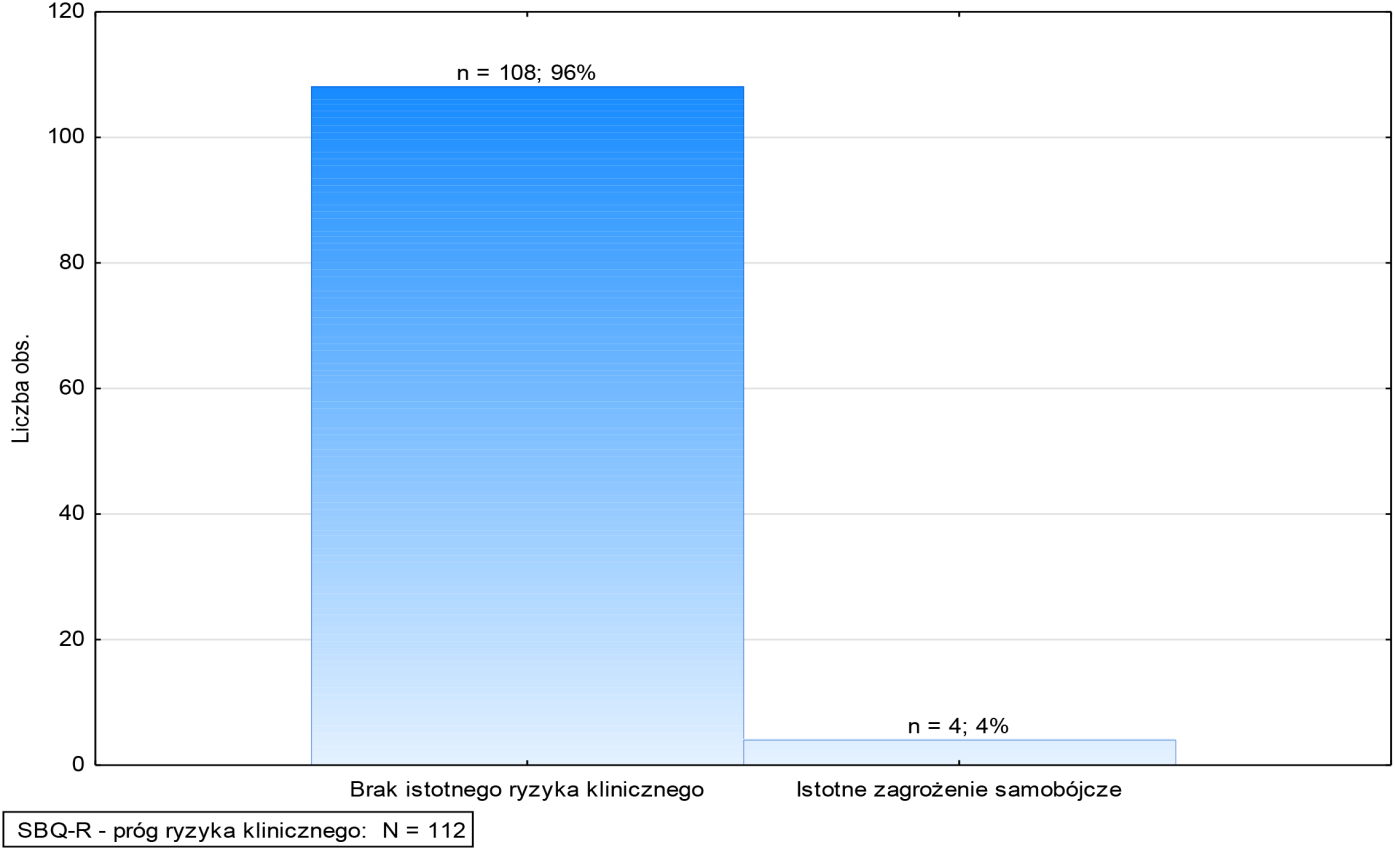
Suicidal Behavior Questionnaire (SBQ-R) – clinical risk threshold (adult patients).

**Figure 3:**
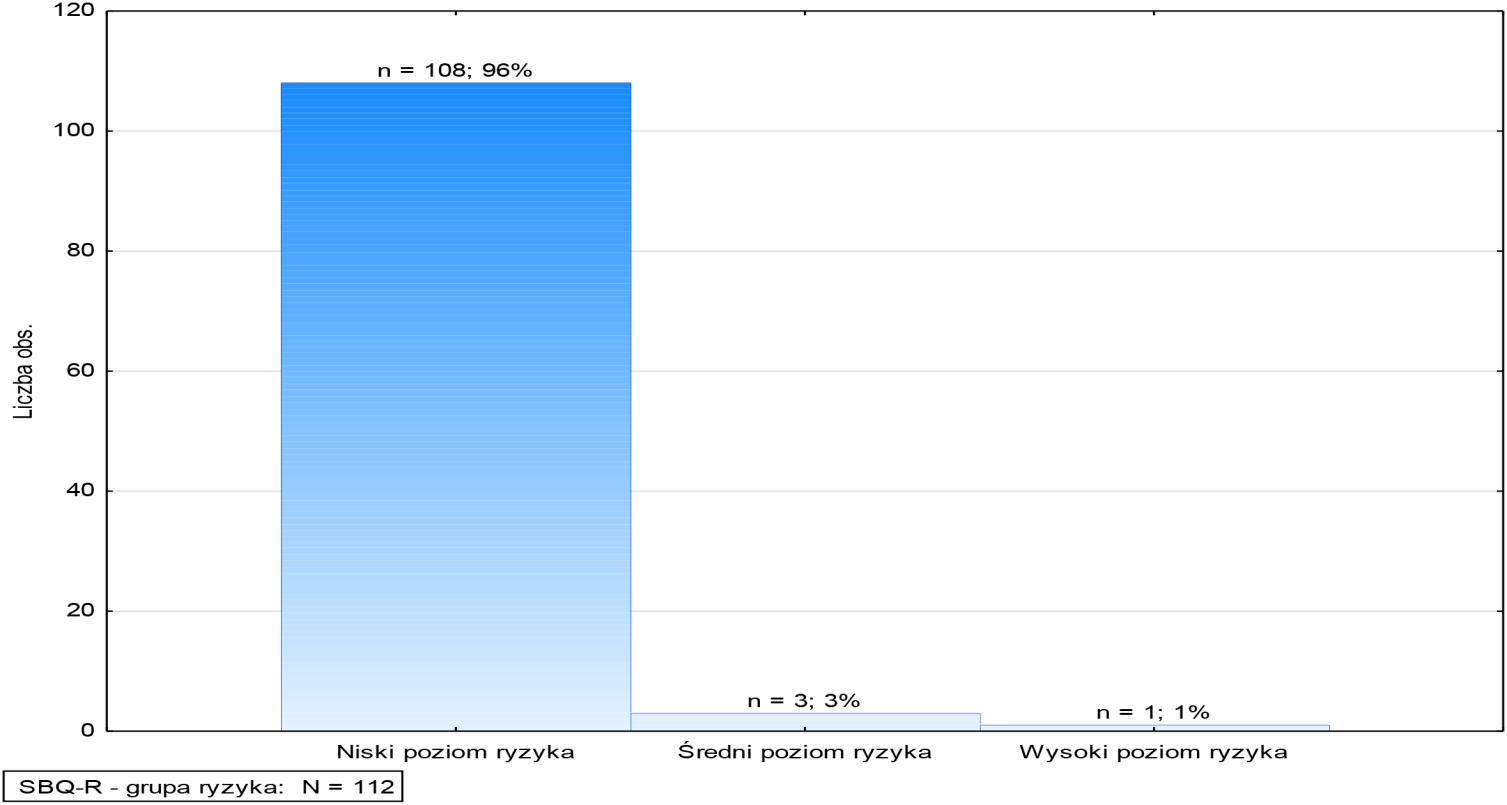
Suicidal Behavior Questionnaire (SBQ-R) – risk groups.

Based on the obtained results, patients were classified into three risk groups (**Figures 2**, **3**):

1. Low risk – 96% (n=108)

The vast majority of patients studied were assigned to the low-risk category, meaning they did not exhibit significant symptoms suggesting suicidal risk. Their scores fell within the lower range of the SBQ-R scale, suggesting that this group was unlikely to attempt suicide.

2. Medium risk level – 3% (n=3)

A small proportion of the study participants were classified as medium-risk, meaning they reported occasional suicidal thoughts or episodes of uncertainty about their lives. Although these were relatively few in number, they warranted close attention and further evaluation to determine whether existing symptoms could escalate to a higher risk level in the future.

3. High risk level – 1% (n=1):

One patient was assigned to the high-risk group, with an SBQ-R score exceeding 12. In this case, immediate, detailed psychiatric evaluation was necessary, as well as implementation of potential interventions, such as intensified supervision, additional consultations, and modification of pharmacological treatment.

Analysis of the relationship between the age of patients hospitalized in forensic psychiatry wards and the scores obtained on the Suicide Risk Assessment Scale (SBQ-R) revealed no statistically significant differences (**Table 2**). The study results indicate that the mean SBQ-R values across age groups are similar, and their medians remain unchanged, suggesting no clear effect of age on the assessed risk level. These observations are confirmed by the Kruskal- Wallis test, in which the obtained value of p = 0.75 (p > 0.05) indicates a lack of statistical significance.

**Table 2:** The relationship between the age of patients hospitalized in forensic psychiatry wards and the results obtained in the Suicide Risk Assessment Scale (SBQ-R) – Kruskal- Wallis test.

Age	SBQ-R - Total Score - descriptive statistics						Wallis test
	(Mean)	(N)	(Min.)	(Max.)	(Standard Dev)​	(Median)	
Up to 30 years	3.42	12	3	5	0.79	3	H (4, N = 112) =1.87 p =0.75
31–40 years old	3.55	33	3	8	1.15	3	
41–50 years old	3.65	31	3	8	1.45	3	
51–60 years old	3.67	12	3	6	0.98	3	
Over 60 years old	3.71	24	3	12	2.07	3	

The length of stay of patients in the forensic psychiatry ward did not significantly affect the SBQ-R scores (p=0.11) (**Table 3**). Mean SBQ-R values in different time groups indicate slight fluctuations, but patients who stayed in the hospital for 5 to 9 years achieved the highest scores (mean 4.73).

**Table 3:** The relationship between the length of stay of patients in the forensic psychiatry ward and the results obtained in the Suicide Risk Assessment Scale (SBQ-R) – Kruskal-Wallis test.

Duration of stay in the ward	SBQ-R - Total Score - descriptive statistics						Wallis test
	(Mean)	(N)	(Min.)	(Max.)	(Standard Dev)​	(Median)	
up to 1 year	3.25	32	3	6	0.67	3	H (4, N = 112) =7.32 p =0.11
from 1 to 3 years	3.61	41	3	8	1.28	3	
from 3 to 5 years old	3.35	17	3	5	0.70	3	
from 5 to 9 years old	4.73	11	3	12	2.72	4	
over 9 years old	3.91	11	3	8	2.02	3	

Regarding the relationship between the age of the study patients and the level of suicide risk measured by the SBQ-R, the estimated effect size (ϵ²) indicated no effect (ϵ² ≈ 0.00), which is consistent with the statistically insignificant result of the Kruskal-Wallis test and indicates a lack of clinically significant differences in suicide risk between age groups. Similarly, in the analysis of the relationship between length of hospitalization and SBQ-R scores, the estimated value of ϵ² ≈ 0.03 indicates a very small effect, with no clinical significance. This result is consistent with the lack of statistical significance of the Kruskal-Wallis test and the limited variability in the level of suicide risk in the study sample.

An additional analysis was conducted in which the “diagnosis” variable was grouped into two categories: schizophrenia and other diagnoses. The Mann-Whitney test used did not show any statistically significant differences in the level of suicide risk measured by the SBQ-R, which confirms that, given the available sample structure, further dividing the subjects according to diagnosis would not allow for obtaining stable and reliable statistical results.

## Discussion

Although numerous studies have described high and rising suicide rates among psychiatric patients, the risk of suicide among forensic hospital patients has not been adequately studied. To the authors’ knowledge, the current study is the first to assess suicide risk in patients admitted and treated in forensic psychiatric wards in Poland. Therefore, it is difficult to relate its results to other studies, although many issues related to the issues studied are addressed in the scientific literature.

Mental disorders are associated with an increased risk of premature death, including suicide (5–7). Numerous attempts have been made to organize knowledge on suicide risk assessment and the prevention of increasing suicidal behavior. Researchers have sought appropriate diagnostic tools to develop the best possible methods for identifying risk factors associated with suicide. Researchers have demonstrated that there are a number of variables that can be considered factors influencing suicidal behavior. These include: previous suicide attempts, various mental health problems and their severity, personality traits such as impulsivity and neuroticism, genetic markers, anxiety, aggressive behavior, substance abuse, and demographic variables (2, 8–10). Analyzing the variables in this study, it can be concluded that they do not constitute a significant factor differentiating their scores on the Suicide Risk Assessment Scale. Similar studies were obtained in a group of outpatients (11). Therefore, assessing the degree of suicide risk became a key issue. The higher the risk, the more advanced protective procedures should be implemented, along with appropriate treatment tailored to the patient’s current mental state and symptoms (12, 13).

A critical perspective on the ability to predict suicide among patients with mental health problems has existed for many years. In the 1980s, Pokorny (14) examined suicide risk using various tools in a group of 4,800 individuals hospitalized in psychiatric hospitals. The results showed that it was impossible to clearly identify specific individuals at increased risk.

Chan et al. (15) analyzed suicide risk among individuals who engage in self-harm and identified four risk factors: previous episodes of self-harm, suicidal intent, physical health problems, and male gender. Clinicians, aware of the difficulties associated with using various indicators to estimate a patient’s likelihood of suicide, often rely on their intuition when assessing this risk. Advances in technology are opening new perspectives, indicating that suicide risk and prevention methods can be supported by modern methods such as mobile phone applications and information technology (16).

However, it is also important to note the existence of scientific publications indicating an increased incidence of suicide among forensic psychiatric patients.

An interesting analysis was provided by an American study comparing the incidence of suicidal behavior over many years. The results of this analysis indicate significant variation in the suicide rate depending on the historical period, with suicides occurring before 1968 being significantly less frequent. In comparison, the post-1968 rate of 232 per 100,000 is comparable to data presented in a series of studies on suicide in psychiatry and significantly higher than that obtained in American prison population studies. The results strongly suggest that forensic hospital populations have suicide rates broadly comparable to other psychiatric populations (17).

Clarke et al. followed 595 patients for up to 20 years and found that 18 of them (3%) successfully committed suicide (18). They noted that these were more often individuals diagnosed with mental illness than with personality disorders. A separate issue that also requires further research is the risk of suicide after leaving a forensic psychiatric ward. Research conducted in Japan indicates that the incidence of suicide deaths in this group is high (19). Suicides accounted for over half of all deaths. Women were a particularly vulnerable group.

A study by Chinese researchers summarizing the prevalence of suicide in this patient group indicates that individuals with serious mental illnesses have a higher risk of suicide than the general population, especially if they exhibit severe aggressive behavior. Evidence suggests that patients in forensic psychiatric wards have a high suicide rate. One study found a suicide rate of 0.2% in a US psychiatric hospital, approximately 13 times the rate for all men in the general US population. A national follow-up study conducted over 29 years in England and Wales found that the suicide rate was 40 times higher for women and almost seven times higher for men in maximum security hospitals than in the general population. Furthermore, many patients continued to be at high risk of suicide after discharge from forensic psychiatric hospitals (20).

Interesting research was also presented by Finnish scientists who analyzed the causes of mortality among patients undergoing compulsory psychiatric treatment in Finnish hospitals between 1980 and 2009 by categorizing the causes of mortality into somatic diseases, suicides, and other unnatural deaths. The causes of death were analyzed in 351 patients who died during hospitalization. The vast majority (249/351) of deaths were caused by somatic diseases, 59 patients committed suicide, four patients were murdered, and 32 deaths were accidental. The results of this study indicate that attention should be paid to the high risk of suicide when organizing inpatient and outpatient treatment for forensic psychiatric patients. Furthermore, the risk of accidents should be assessed and patients should be provided with appropriate somatic care during forensic psychiatric treatment, as well as its continuation in the outpatient setting (21).

Patients in forensic psychiatry wards are under complete surveillance, which may blind them to the real possibility of suicidal ideation. Some patients may be reluctant to openly admit to suicidal thoughts to avoid additional supervision (22).

From the patient’s perspective, not admitting to suicidal thoughts can be a rational adaptive strategy. Reporting such thoughts can therefore result in constant surveillance, limited privacy, isolation, and delayed treatment progression or discharge (23).

Patients make an informal calculation, where the costs of disclosure include stigma, loss of control, and a more stringent regimen, while the benefits of concealment are associated with greater autonomy and a “better clinical picture” (24, 25). In a court setting, this calculation is particularly intensified, as clinical decisions have real legal consequences.

Concealing suicidal ideation in forensic psychiatry is not a “lack of cooperation” on the part of patients, but a logical response to the structure of the system, in which safety is achieved through control. This problem reveals the tension between the protection of life, the effectiveness of therapy and respect for the patient’s subjectivity (26).

Without taking these contradictions into account, subsequent surveillance procedures may paradoxically increase rather than reduce the very risk they are intended to mitigate. In such studies, patients may consciously minimize their responses, especially in the context of psychiatric hospitalization, meaning the actual severity of some symptoms may be underestimated.

Despite the fact that forensic psychiatric patients are in a very difficult situation characterized by years of isolation, detachment from the external environment, and limited autonomy, the results of most patients’ studies are in the minimal range, suggesting a lack of significant suicidal risk.

Researchers also point out that there is a lack of specific therapies addressing the problem of suicide, and there is no convincing evidence of their effectiveness (27). However, implementing programs focused on providing support to individuals who may experience suicidal ideation is important from the perspective of clinical effectiveness (12, 28, 29).

This study is innovative. For the first time, the value of monitoring suicide risk in patients in forensic psychiatry departments was addressed using a simple questionnaire. The results suggest the need to introduce such monitoring into routine clinical practice in such departments. Identifying suicide risk, especially in patients with longer stays, may necessitate the implementation of psychological and pharmacological interventions that would support emotion regulation and reduce feelings of isolation among forensic psychiatry patients.

Due to the fact that forensic psychiatry patients suffer from severe mental disorders, have a history of violence, experience prolonged confinement, and legal uncertainty, it can be assumed that globally, this is one of the most vulnerable groups in terms of suicide. Therefore, risk assessment is significant and worth comparing with other countries. Thanks to such procedures, suicide risk assessment is becoming a component of international standards for psychiatric care, and its quality allows for comparisons between healthcare and justice systems and the identification of good and bad practices on a global scale.

The study utilized older bibliographic sources, as the authors believe they constitute an important element of scholarly work. The cited sources not only provide insight into the origins of concepts, theories, and research methods, but also facilitate understanding the historical context of scientific development and assessing continuity and change. Citing older works on this topic increased the reliability of the study and prevented the duplication of existing results.

The study has numerous limitations. The results come from a group of patients treated at a single forensic psychiatry center, albeit in different wards. To draw general conclusions, it is necessary to compare these results with others from other centers.

A limitation of the study was that it was conducted in a male population, which resulted from the specific characteristics of the centers where the study was conducted. The gender limitation makes generalization difficult, especially considering possible gender differences in the expression of emotions and coping with solitary confinement. It would be interesting to compare them with studies in a group of women.

The study used questionnaires whose reliability is difficult to verify and is based on the assumption of respondent truthfulness. Relying on such tools in psychotic populations raises significant issues with the validity of the obtained data. Individuals with psychotic symptoms often experience cognitive deficits that may hinder their ability to properly understand questions, assess their own emotional states, and provide consistent answers. Additionally, the variability of insight—typical of psychotic disorders—means that their awareness of the illness and their ability to objectively assess themselves can fluctuate significantly over time. As a result, responses may be unstable, less coherent, and subject to distortions, limiting their diagnostic and research usefulness.

Another limitation of the study is the use of a single self-assessment tool, without triangulation with other methods, such as clinical interviews or behavioral observations. This may affect the validity of the obtained results. Future studies should incorporate a multi-source approach to measuring the studied construct and explore longitudinal designs to distinguish the effects of initial adaptation, long-term incarceration, and other covariates, such as diagnosis, type of pharmacotherapy, and prior forensic stays.

The study also did not consider reactive factors, such as the influence of the type of offense committed on mood perception.

Another methodological limitation of the study was the possibility of response bias. Patients’ responses may have been biased due to the possibility of changes in treatment or increased supervision as a result of disclosing information about their suicidal ideation.

## Conclusions

Long-term stays in forensic psychiatric wards among men with mental illness are associated with a low risk of suicide. This risk does not appear to be related to patient demographics. This is a pilot study, and the findings require confirmation in subsequent patient groups.

## Data Availability

The raw data supporting the conclusions of this article will be made available by the authors, without undue reservation.
